# Compassionate Leadership: Essential for the Future of Tropical Medicine and Global Health

**DOI:** 10.4269/ajtmh.21-0832

**Published:** 2021-10-11

**Authors:** Evan Harrel, Laura Berland, Julie Jacobson, David G. Addiss

**Affiliations:** ^1^ Center for Compassionate Leadership, Montauk, New York;; ^2^ Bridges to Development, Seattle, Washington;; ^3^ Focus Area for Compassion and Ethics, Task Force for Global Health, Decatur, Georgia

## Abstract

Compassion—the awareness of suffering coupled with the desire to relieve that suffering—is an evolved human capacity that offers significant benefits for individuals and organizations. While the relief of suffering is central to tropical medicine and global health, compassion is more often assumed than explicit. Global health leaders participating in a compassionate leadership program recently reported that the most common personal barriers to compassionate leadership include inability to regulate workload, perfectionism, and lack of self-compassion; while the most common external challenges include excessive work-related demands, the legacy of colonialism, and the lack of knowledge on how to lead with compassion. These barriers can be surmounted. Within organizations, leaders are the primary shapers of compassionate cultures. Now is the time to bring our core compassionate values to bear in addressing the “unfinished business” of ensuring global health equity and deconstructing colonialist structures in global health and tropical medicine. Compassionate leadership offers us tools to complete this unfinished business.

## INTRODUCTION

Compassion—the awareness of suffering coupled with the desire to relieve that suffering^1^—is more often assumed than explicit in the fields of tropical medicine and global health. It is not that compassion is absent. In fact, it is very much alive, serving as a fundamental source of motivation for those who enter these fields.^2^ In her 2017 Presidential address to the American Society of Tropical Medicine and Hygiene (ASTMH), Patricia Walker included compassion as a core global health value.^3^ In addition, influential leaders in tropical medicine are often remembered or praised for their compassion.^4^^,^^5^

However, compassion lies largely beneath the surface of our collective discourse. In busy day-to-day practice, especially in public health, compassion is often lost. We lose sight of the individual human beings who are affected by our population-level health interventions. As in healthcare more broadly, the cost to practitioners, organizations, and “beneficiaries” of a lack of compassion is immense.^6^

Recent events have prompted an urgent reexamination of compassion in tropical medicine and global health. These include: 1) the profound injustices revealed and exacerbated by COVID-19, including the epidemics of burnout and moral injury among caregivers, and the pandemic’s impact on essential health services^7^; 2) the escalating demand for global health and tropical medicine to confront their colonial past; and 3) the identification of ‘compassion’ as a key theme for the Society by its current President.^8^

## COMPASSION SCIENCE

Compassion is a human capacity that evolved in the context of early forms of community living. In *The Descent of Man*, Charles Darwin noted the selective advantages of responding to suffering with care and concern.^9^ The social benefits of compassion are increasingly well-documented. Within organizations, greater levels of compassion lead to better financial performance, higher employee and customer retention, and executive perception of improved effectiveness.^10^ Compassion also can contribute to greater creativity, innovation, and sustainable strategic advantage.^11^ Within healthcare in particular, compassion has significant, positive effects on quality of care and clinical outcomes for patients,^12^ and on provider well-being, including reduced burnout.^13^

Recent insights from psychology and neuroscience suggest that compassion is comprised of at least three fundamental elements: 1) awareness of the suffering of others, 2) empathic understanding and concern for others, and 3) action to alleviate the suffering.^14^ In tropical medicine and global health, awareness of suffering arises both from individual patient encounters and from more abstract population-level data. An understanding of only the latter may not always be sufficient to generate adequate empathy, a critical pathway to compassionate action.

The terms empathy and compassion are sometimes used interchangeably, but neuroscience has distinguished the neural pathways of empathy from those of compassion. The difference is important. Awareness of suffering may lead to empathic distress, which activates the pain centers in the brain. In contrast, moving from empathy to compassionate action activates the reward regions of the brain,^15^ as well as the motor cortex.^16^ We derive pleasure and meaning not just from empathetic understanding but from compassionate action—and even from observing acts of compassion.^17^

## BARRIERS TO COMPASSIONATE LEADERSHIP

Given the many benefits of compassion for individuals and organizations, as well as its relevance to global health and tropical medicine, what holds us back from acting compassionately? Of 45 global health leaders from 15 countries who participated in our recent courses on Compassionate Leadership and Resilience,^18^ 33 responded to survey questions about personal and organizational barriers that constrain their compassion. Primary personal or internal challenges include inability to regulate workload (70%), perfectionism (70%), and lack of self-compassion (the offering of compassion to oneself when suffering) (61%), while the most common external challenges include excessive work-related demands (76%), the legacy of colonialism (52%), and lack of knowledge about how to consistently act and lead with compassion (46%).

Inability to regulate workload and excessive work-related demands represent internal and external manifestations of the same challenge. Early research recognized that the perception of not having enough time limits our capacity to respond to suffering with compassion.^19^ Excessive workload and the perceived inability to control it is also a serious cause of burnout.^20^ Particularly in context of the global COVID-19 pandemic, burnout is a growing concern.^21^

The barriers of perfectionism and lack of self-compassion are related as well. Lack of self-compassion often leads to perfectionism, in which failure to adhere to unrealistic personal or professional standards is accompanied by harsh criticism of self and others.^22^ One form of perfectionism, “compulsion to save the world,” is particularly common among global health professionals.^2^ These behaviors contribute to pathological altruism,^23^ in which action undertaken to benefit others instead results in harm or unintended negative outcomes.^24^

Colonialism has fueled many unintended negative outcomes and caused immense social suffering. As john a. powell notes, social suffering is optional; it is the “result of our social arrangements” rather than inherent in the process of living, growing old, and dying (“ontological suffering”).^25^ Tropical medicine and global health evolved and advanced in the wake of colonialism. As such, they contribute to social suffering, often in ways that may not be apparent. Compassionate leadership is not about acquiescence, but rather, resolutely working toward—and demanding—the end of systemic injustices and inequities that perpetuate social suffering. It is about realizing the “solidarity dividend” that comes through creating deeper human connections among all of us.^26^

Leaders in global health and tropical medicine are not alone in lacking knowledge about how to lead with compassion. Ninety-one percent of more than 1,000 business leaders surveyed said that compassion was very important, and 80% said they would like to strengthen their compassion skills, but don’t know how.^27^ Fortunately, this barrier can be overcome. Compassion is innate: children as young as 20 months show evidence of compassion skills.^28^ Compassion is also trainable. In adults, compassion training reinforces neural circuits of the brain that are activated with compassion and lead to more compassionate behavior.^29^

## OVERCOMING BARRIERS

In the context of a suffering world, given the strong commitment of those in global health to alleviate that suffering, what hope do we have of reducing these barriers? Effective responses include mindfulness, self-compassion, and organizational cultures that promote compassionate responses to suffering.

Mindfulness—slowing down on purpose, to pay attention, and focus one’s awareness in the moment nonjudgmentally^30^—can help address the challenges of time demands, although this may initially appear counterintuitive. Benefits of mindfulness that promote compassion include reduced rumination and emotional reactivity,^31^ lower stress and anxiety,^32^ and less frequent impulsive action.^33^ Mindfulness allows for greater awareness of the suffering of others, a less-distorted understanding as to the causes of that suffering, and time-effective engagement in actions to transform it.

Self-compassion is the offering of compassion to oneself when we experience suffering. It involves the same elements as other-directed compassion: awareness, empathy, and action, although the focus is on the self rather than someone else.^34^ As previously noted, self-compassion directly moderates the barrier of perfectionism, reported by 70% of respondents in our survey. Self-compassion helps to transform perfectionism into conscientiousness.^22^

Individual training in self-compassion and mindfulness will not, in itself, address systemic barriers to organizational compassion, which aligns closely with the culture of quality healthcare promoted by WHO.^35^ Compassionate organizational culture is primarily shaped by leaders who are skilled in compassionate communication, creating psychological safety, and conveying a sense of belonging for all. Psychologically safe work environments, in which people can speak up without fear of punishment, not only foster compassion; they also reduce employee stress and anxiety^36^ and improve team effectiveness.^37^ Compassionate communication allows leaders to use every communication opportunity to promote cohesion and growth while avoiding fear, guilt, and shame.^38^ Creating a culture of belonging—imperative for the future of global health—promotes equity and relieves the stress of exclusion.^39^ Opportunities for leaders to develop and practice these skills are increasingly available.^11^^,^^18^^,^^22^^,^^40^

## COMPASSIONATE LEADERSHIP: COMPASSION, COURAGE, AND CULTURE

Given the crescendo of calls to “decolonize” global health, the devastating toll of COVID-19 on healthcare and public health systems, and increasing polarization of society, the need for ASTMH members, individually and collectively, to lead with compassion has never been greater. Julie Jacobson has highlighted three key themes during her term as President of the Society: compassion, culture, and courage. Compassionate leadership is relevant to all three.

As noted, compassion is a core global health value. Alleviating suffering and its causes is a fundamental purpose of global health and tropical medicine. Realizing this purpose requires compassionate individuals, organizations, and systems, which in turn depend on skillful, compassionate leadership.

Compassionate leadership also requires courage, as it seeks to address the root causes of suffering. Trying to alleviate suffering without addressing its root causes contributes to the incessant work-related demands experienced by so many in global health. When people consider compassion, they often think of its nurturing features. But, compassionate action—particularly to remove the root causes of suffering at the organizational or systems levels—is often courageous. Joan Halifax describes these two aspects of compassion—nurture and courage—as its soft front and strong back.^41^ Both are needed and both must be cultivated.

Global health has enabled remarkable achievements in disease control and health promotion. Now is the time to address our “unfinished business” of ensuring global health equity, ending colonialist attitudes and structures and reversing their negative impacts, embracing and honoring our cultural differences, and doing this in ways that create day-to-day work environments that reflect our core compassionate values. Compassionate leadership offers us the tools to complete this unfinished business and bring us into a future where global health and tropical medicine reach their full potential in service of a resilient world.
